# S-adenosylmethionine (SAM-e) for the treatment of depression in people living with HIV/AIDS

**DOI:** 10.1186/1471-244X-4-38

**Published:** 2004-11-11

**Authors:** R Andrew Shippy, Douglas Mendez, Kristina Jones, Irene Cergnul, Stephen E Karpiak

**Affiliations:** 1 ACRIA (AIDS Community Research Initiative of America), 230 West 38^th ^St, 17^th ^Floor, New York, NY 10018, USA

## Abstract

**Background:**

This study reports on clinical data from an 8-week open-label study of 20 HIV-seropositive individuals, diagnosed with Major Depressive Disorder (DSM-IV), who were treated with SAM-e (S-Adenosylmethionine). SAM-e may be a treatment alternative for the management of depression in a population reluctant to add another "pill" or another set of related side effects to an already complex highly active antiretroviral therapy (HAART) regimen.

**Methods:**

The Hamilton Rating Scale for Depression (HAM-D) and the Beck Depression Inventory (BDI) were used to assess depressive symptomatology from 1,2,4,6 and 8 weeks after initiation of treatment with SAM-e.

**Results:**

Data show a significant acute reduction in depressive symptomatology, as measured by both the HAM-D and the BDI instruments.

**Conclusions:**

SAM-e has a rapid effect evident as soon as week 1 (*p *< .001), with progressive decreases in depression symptom rating scores throughout the 8 week study.

## Background

The incidence of depression in people living with HIV (PLWH) has consistently been reported to be higher than the 12–15% rates reported for the general population [1]. Depression in this population is largely untreated. Estimates suggest that this comorbid condition of HIV affects 10% – 50% of PLWH [2]. The management of depression in people with HIV is important since it can reduce medication adherence. Depression can be life-threatening, given that the patient can deteriorate medically, and that untreated depression can result in suicide. Untreated depression may also result in substance abuse, a theory based on the 'self medication' theories of addictive behavior. Depression is a pressing problem at this point in the epidemic because the HIV population is aging and the incidence of depression increases with age [3].

In New York City, 27% of all people living with HIV are over 50 years of age and 66% are over 40 years age [4]. In two studies of PLWH age 50 and older, the rate of depressive symptomatology ranged from 58% – 68% [5].

Under-treated depression in the HIV population may be the result of multiple factors. People of color, women, substance abusers, and those living in poverty are less likely to have access to or to accept mental health outpatient services [5-7]. Many patients with HIV who are managing their disease are on complex regimens of medications (HAART) and dietary supplements to treat their illness [8]. Adding another pill with significant side effects is often viewed by both patient and primary treating physician as an unacceptable burden, either in terms of number of pills per day, or in terms of anticipated side-effect profiles, or risk/benefit analysis [9]. Primary care physicians are often unprepared to recognize depression, which can present as fatigue, weakness, insomnia or loss of appetite, and may seek medical explanations for depressive symptoms [7]. HIV-related dementia, which presents with apathy and amotivation, can also be mistaken for depression. Physicians may be unfamiliar with mental health interventions and place depression as a lower priority after management of the primary HIV infection [10]. The stigma of HIV remains large and the addition of the potential stigma of a mental illness (i.e. depression) further contributes to the patient's reluctance to pursue or engage treatments for depression [11]. Many patients fear that substance abuse, homosexuality, or HIV itself may be grounds for discrimination from a mental health provider [12,13].

SAM-e is a compound that has been used for many years in Europe as an antidepressant. It became available in the USA as a non FDA-regulated compound in 1999. SAM-e resembles a naturally occurring compound in the human body, formed by methionine and adenosine triphosphate. SAM-e donates an active methyl group to several acceptor molecules, including catecholamines, fatty acids, phospholipids, proteins and nucleic acids [14]. SAM-e appears to increase central turnover of dopamine and serotonin; for example it increases the main metabolite of central serotonin-5-HIAA (5-hydroxyindoleacetic acid) in the cerebrospinal fluid [15].

The efficacy of SAM-e as an antidepressant has been reported in a number of open-label trials [16] and in two small placebo-controlled clinical trials [17,18]. Kagan et al conducted a placebo-controlled trial that found SAM-e to be effective in the treatment of depressed inpatients [17]. A meta-analysis of SAM-e studies shows a greater response rate with SAM-e compared with placebo, with a global effect size ranging from 17–51% [19]. Side effects reported in these studies are fewer than with SSRIs specifically with respect to nausea, headache, dry mouth, and agitation or activation. In studies of parenteral SAM-e, activation of mania was noted in two cases [20]. SAM-e would seem to offer the potential for effective antidepressant treatment with fewer side effects for patients with medical illnesses. This was shown in a successful open label trial of SAM-e in Parkinson's patients [21] and in a double-blind study of fibromyalgia patients [22]. The aim of the study reported below was to assess efficacy, tolerability, and safety of SAM-e in a group of HIV-positive individuals diagnosed with Major Depressive Disorder (DSM-VI).

## Methods

This study is an analysis of the complete dataset from an independently funded, IRB-approved project conducted at ACRIA, a 12-year-old, community-based HIV research and education agency in New York City. A preliminary report, based on a subset of these data, was presented at the 14^th ^International Conference on AIDS. A total of 20 patients were recruited between April 2000 and November 2000.

Recruitment activities included distribution of information brochures to community physicians, announcements at public ACRIA treatment education workshops, and direct calls to persons listed in the ACRIA database. Individuals who responded to recruitment materials were screened via phone by the research coordinator using the PRIME-MD [23]. HIV-positive patients were then invited to be interviewed by a study psychiatrist at ACRIA for a clinical psychiatric examination. Patients who met DSM-IV criteria for Major Depression (assessed with the SCID-IV) and who did not meet any of the exclusion criteria were eligible for participation after giving informed consent [24]. Criteria for exclusion were (a) unstable medical illness, (b) pregnancy, lactation or refusal by participants to employ an acceptable contraceptive, (c) history of substance abuse in the prior month, (d) treatment with another psychotropic medication within two weeks prior to initiation of SAM-e treatment, (e) concurrent MAOI treatment, (f) active suicidal ideation and/or psychotic symptoms, (g) reversible medical pathology that is thought to be causing the depression, and (h) history of mania or diagnosed bipolar disorder. Patients received $15 for each completed study visit.

Treatment was initiated at 200 mg of SAM-e twice a day with daily supplementation of 1,000 mcg Vitamin B12 and 800 mcg Folic Acid, which were provided by Pharmavite, LLC. Over the course of the study, the dose of SAM-e was individually adjusted up to 800 mg, twice daily. Patients reporting nausea or insomnia were not increased above 800 mg daily, while patients with no reported side effects could be increased to a total daily dose of 1600 mg. Dosing was adjusted according to the severity of symptoms and clinical treatment response. We defined clinical treatment response as an improvement in depressive symptomatology of greater than 50% reduction on patient scores on the BDI and HAM-D as our treatment endpoint [25,26]. A statistical data codebook was created and secondary statistical analyses were performed.

### Measures

At each study visit (Baseline, Week 1, Week 2, Week 4, Week 6, and Week 8), patients completed a clinical interview and a series of structured questionnaires on medical/physical symptoms, substance abuse, dementia scales, as well as two instruments designed to measure depressive symptoms, self-administered Beck Depression Inventory (BDI and the clinician-administered HAM-D).

#### BDI

The BDI is a self-report measure for depressive symptoms [25]. Total scores range from 0 – 63 and are calculated by summing the scores to each of the 21 items. Higher scores on this measure indicate greater severity of depression. Scores above 30 indicate severe depression. Scores of 30 – 10 describe moderate depression, while scores less than 10 indicate the absence of depression.

#### HAM-D

The HAM-D is a 17-item, clinician-rated instrument. It was designed to assess the changes in severity of depressive symptomatology over time in patients who had been diagnosed with Major Depressive Disorder [26]. Scores above 24 reflect severe depression, while scores ranging from 17 – 7 indicate mild symptomatology. Scores below 7 indicate an absence of depression.

### Analyses

Two separate analyses (e.g., Baseline -v- Week 1 and Baseline -v- Week 8) were identified as the most critical indicators of SAM-e efficacy. The first analysis represented evidence for acute onset of SAM-e, and the second indicated effectiveness of SAM-e over time. BDI and HAM-D scores were centered and the resulting Z-scores were used in a series of t-tests to assess the agreement between patient and investigator ratings of depression. Mean scores from both depression measures (BDI and HAM-D) were compared between time points, using a series of paired t-tests. Finally, an intent-to-treat analysis, using the last score carried forward, was conducted on patient BDI scores for all subjects who initiated treatment.

## Results

### Participants

Thirty (30) subjects were screened by telephone and twenty (20) entered into the study. Of these, fifteen (15) were male, five (5) were female and the majority were people of color (50% Black, 30% Hispanic, 20% Caucasian). Table 1 presents a detailed demographic profile of the patients. Although the exclusion criteria included a prohibition against concurrent treatment with conventional antidepressants during the study period, we identified one patient who did not discontinue use of conventional antidepressant until Week 4 of the study. All subjects self-reported that they were not actively substance abusing at the time of the study; however, urine drug screens were not performed. Five (5) patients did not complete the study, but Week 1 data were recorded and were included in the analysis of early onset of therapeutic SAM-e effects. These five (5) subjects were excluded from subsequent analyses. Two (2) subjects did not return for study participation after Week 1: both had a history of IDU; one was co-infected with Hepatitis C and had a comorbid obsessive compulsive disorder (OCD). Three (3) patients did not return after Week 4, one with a history of IDU and one with a history of suicide attempts. Of those who dropped out of the study, two (2) met criteria for AIDS.

**Table 1:** Demographic characteristics of patients

Variable	*N*	%	Median	Range
Age	20		45	24 – 57
Sex				
Male	15	75.0		
Female	5	25.0		
Race/Ethnicity				
Black	10	50.0		
Hispanic	6	30.0		
Caucasian	4	20.0		
HIV diagnosis (year)	20		1992	1987 – 2000
CD-4 count (baseline)	20		320	5 – 1200
Log viral load (baseline)	20		3.40	1.70 – 4.48
Transmission risk				
MSM sex	4	20.0		
Heterosexual sex	5	25.0		
IDU	2	10.0		
Multiple risks	9	45.0		

### Treatment response

Table 2 contains the means, standard deviations and ranges for depression data from each study visit. Of the patients who provided Baseline and Week 1 follow-up data, there was clear evidence for a rapid therapeutic effect of SAM-e. The reduction in mean BDI scores from Baseline (*M *= 33.5, *SD *= 11.1; Range: 15 – 55) to Week 1 (*M *= 18.9, *SD *= 10.4; Range: 0 – 45) was significant, *t*(1, 18) = 5.15, *p *< .001. Mean HAM-D scores declined in a parallel manner, from Baseline (*M *= 26.5, *SD *= 6.8; Range: 12 – 39) to Week 1 (*M *= 16.8, *SD *= 7.3; Range: 2 – 29). This change was also statistically significant, *t*(1, 14) = 3.58, *p *< .01. Both depression assessment instruments reached the defined clinical treatment response threshold of a greater than 50 percent reduction in depression symptom scores.

**Table 2:** BDI and HAM-D scores recorded at each study visit

	**BDI**			**HAM-D**		
**Study Visit**	***N***	***M(SD)***	**Range**	***N***	***M(SD)***	**Range**

Baseline	20	33.5(11.1)	15–55	20	26.5(6.8)	12–39
Week 1	19	18.9(10.4)	0–45	15	16.8(7.3)	2–29
Week 2	17	14.1(8.2)	0–25	13	10.7(5.5)	0–21
Week 4	17	8.8(7.8)	0–28	15	6.0(4.7)	0–15
Week 6	16	6.4(6.8)	0–20	14	5.2(5.7)	0–20
Week 8	16	5.0(4.7)	0–16	15	3.7(3.3)	1–13

The 15 patients who completed the study also demonstrated clinical treatment response over time. Mean BDI scores dropped significantly from Baseline (*M *= 35.1, *SD *= 12.0; Range: 15 – 55) to Week 8 (*M *= 5.1, *SD *= 4.8; Range: 0 – 16), *t*(1, 14) = 8.28, *p *< .001. Similarly, HAM-D scores were reduced from Baseline (*M *= 26.7, *SD *= 6.3; Range: 12 – 36) to Week 8 (*M *= 3.7, *SD *= 3.3; Range: 1 – 13). The reduction in HAM-D scores reached statistical significance, *t*(1, 13) = 9.92, *p *< .001.

**Figure 1:**
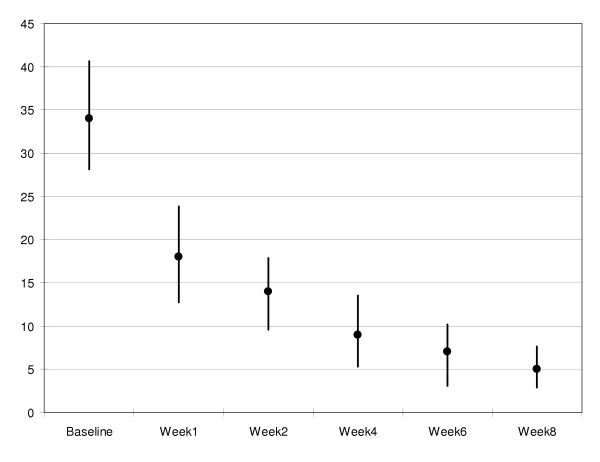
Mean BDI scores at each study visit (95% confidence intervals)

**Figure 2:**
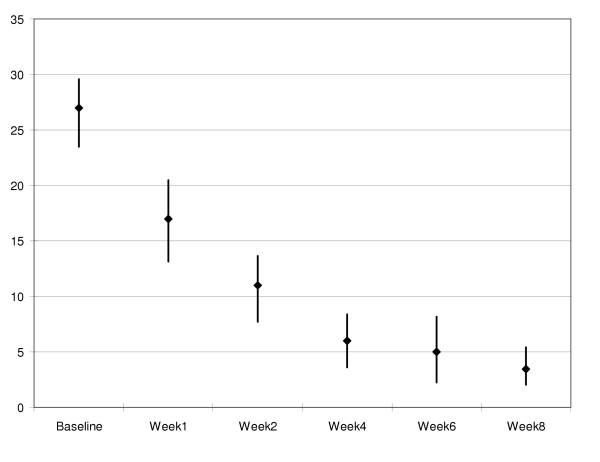
Mean HAM-D scores at each study visit (95% confidence intervals)

There were no significant differences in patient and physician ratings of depression at each study visit (Table 3). Figures 1 and 2 provide graphic representations of mean BDI and HAM-D scores across each time point, respectively. Remission of depression was defined as a HAM-D score ≦ 7, response to treatment was defined as ≧ 50% decrease in HAM-D scores. For the 15 patients who completed the study, the remission rate was 93%. Fourteen (14) of the 15 patients achieved a HAM-D of 7 or lower, while one patient received a HAM-D rating of 13. The response rate was also 93%, with 14 of the 15 patients achieving 50% or greater reduction in HAM-D scores. The patient who did not meet the response rate had a Baseline HAM-D of 12 and a Week 8 score of 7.

**Table 3:** Comparison of BDI and HAM-D scores (study completers)

**Study Visit**	***t***	***df***	***p***
Baseline	-0.07	14	.95
Week 1	-1.39	14	.19
Week 2	-0.98	14	.35
Week 4	-0.64	14	.54
Week 6	0.35	13	.74
Week 8	0.71	14	.49

The intent-to-treat analysis yielded similar results. The last reported BDI and HAM-D scores for each of the 20 patients who initiated treatment (i.e., received at least one dose of SAM-e) were used in this analysis. Baseline BDI scores (*M *= 33.5, *SD *= 11.1; Range: 15 – 55) were higher than Week 8 (*M *= 6.6, *SD *= 6.1; Range: 0 – 21). HAM-D scores dropped from Baseline (*M *= 26.5, *SD *= 6.8; Range: 12 – 39) to Week 8 (*M *= 7.7, *SD *= 10.1; Range: 1 – 39). The reduction in self-reported (i.e., BDI) depressive symptomatology was significant, *t*(1, 19) = 8.85, *p *< .001. The results were equivalent for the psychiatrist-rated HAM-D, *t*(1, 18) = 7.23, *p *< .001.

The intent-to-treat remission rate was 79%. Fifteen (15) of the 20 patients achieved a HAM-D of 7 or lower, while one patient did not receive a valid HAM-D rating and four (4) received HAM-D ratings greater than 7 (13, 22, 24, 39, respectively). The intent-to-treat response rate was 74%, with 14 of the 19 patients achieving 50% or greater reduction in HAM-D scores.

### Adverse effects

No patients terminated their participation due to side effects. Two (2) patients reported nausea and one (1) reported diarrhea; all resolved spontaneously. One (1) patient had insomnia and a subjective feeling of 'high energy,' but did not meet criteria for hypomania.

## Discussion

Recent data from the HIV Cost and Services Utilization Study (HCSUS) indicate that almost half the nation's adult HIV patients suffered from symptoms of mental disorders. The report indicates that the prevalence of mental disorders range from four to eight times higher than those found in the general populations. Over 60 percent of HIV-positive adults used mental health or substance abuse services during the period studied; 26% visited mental health specialists, 15% had group mental health treatment, 40% discussed emotional problems with medical practitioners and 30% used psychotherapeutic medications [27].

Depression remains under-reported and under-treated in the HIV population. Patient factors include stigma, fear of more medication, and perhaps the assumption that depression is a normal part of HIV disease, or due to HAART. Physicians tend to focus more on physical symptoms, and may be unaware of how to screen for depression in this population. Both physicians and patients are disadvantaged in accessing gay-affirmative, well-trained HIV mental health specialists who can disentangle HIV medical problems from substance abuse and mental illness. Risk factors for depression (e.g., history of substance use/abuse, lack of social support, stigma, etc.) are prevalent in those populations who are disproportionately affected by HIV. Many patients are reluctant to add more medication to their complex treatment regimens. The need for effective and safe treatments for depression is clear.

Results of this study demonstrate that SAM-e significantly reduces depression in people living with HIV. This finding supports previous research demonstrating the efficacy of SAM-e for use in treating depression. More importantly, the therapeutic effect of SAM-e had an acute onset. There were significant reductions in the severity of depression among study patients within the first week of treatment. Unlike most antidepressants that require several weeks to reach maximum therapeutic efficacy, previous research has shown that serum levels of SAM-e peak within 24 hours of treatment. This may account for the rapid, effects of SAM-e seen on the HAM-D and BDI scores.

## Conclusions

SAM-e is a safe, effective treatment for depression among people living with HIV. SAMe has few reported side effects, and in our population of PLWH, was extremely well tolerated. SAM-e in this group had rapid onset of clinical efficacy. Both of these results make it an appealing alternative therapy for the management of depression in the HIV population.

The results of the current study must be interpreted in the context of the study's limitations. Limitations of this study include the lack of a placebo group, the lack of a double-blind design and small sample size. The criteria for participation and the small sample size may limit the generalizability of our findings to other patient populations. Without a placebo control group, we cannot assess two factors that might have affected our results. Although patients were treatment-experienced with other anti-depressants, we cannot rule out the potential placebo-response that may have contributed to the rapid therapeutic effect of SAM-e. Also, meeting with a study psychiatrist may have contributed to improved depressive symptom ratings. Nonetheless, the study results provide useful and clinically relevant information for treating depression in HIV-positive individuals. Future studies employing a double-blind, placebo-control design are warranted.

The absence of depression does not imply wellness. Many people living with HIV/AIDS report feelings of sadness and depression but do not receive treatment for depression. Future studies should also assess health-related quality of life. Such protocols would determine if people with significant, but subclinical depressive symptomatology would benefit from SAM-e.

## Authors' contributions

RASrecoded study data, conducted statisticalanalyses andco-wrote the final manuscript. DM provided details of the study design and procedures andwas responsible for extractingclinical data from patient records. IC and KJ served as clinical investigators and provided valuable comments on the final paper. SEKwrote the first draft of the paper and co-wrote the final manuscript. All authors read and approved the final manuscript.

## Pre-publication history

The pre-publication history for this paper can be accessed here:


